# Meat Production and Supply Chain Under COVID-19 Scenario: Current Trends and Future Prospects

**DOI:** 10.3389/fvets.2021.660736

**Published:** 2021-05-07

**Authors:** Muawuz Ijaz, Muhammad Kashif Yar, Iftikhar Hussain Badar, Sher Ali, Md. Shafiqul Islam, Muhammad Hayat Jaspal, Zafar Hayat, Aneeqa Sardar, Sana Ullah, Denise Guevara-Ruiz

**Affiliations:** ^1^Department of Animal Sciences, College of Veterinary and Animal Sciences Jhang, University of Veterinary and Animal Sciences, Lahore, Pakistan; ^2^Department of Meat Science and Technology, Faculty of Animal Production and Technology, University of Veterinary and Animal Sciences, Lahore, Pakistan; ^3^Chinese Academy of Agricultural Sciences, Beijing, China; ^4^Department of Animal Sciences, College of Agriculture, University of Sargodha, Sargodha, Pakistan; ^5^Punjab Livestock and Dairy Development Department, Lahore, Pakistan; ^6^Department of Animal and Food Sciences, Texas Tech University, Lubbock, TX, United States

**Keywords:** COVID-19, meat sector, supply chain, price fluctuation, meat production

## Abstract

The COVID-19 pandemic impacted meat production, supply chain, and meat prices that caused a severe socio-economic crisis worldwide. Initially, meat and meat products' prices increased due to less production and increased demand because of panic buying. Whereas, later on, both meat production and demand were significantly decreased due to lockdown restrictions and lower purchasing power of the consumers that results in a decrease in meat prices. In early April 2020, meat packing facilities started to shut down due to the rapid spread of the COVID-19 virus among workers. Furthermore, meat producers and processors faced difficulty in harvesting and shipment of the products due to lockdown situations, decrease in labor force, restrictions in movement of animals within and across the country and change in legislation of local and international export market. These conditions adversely impacted the meat industry due to decrease in meat production, processing and distribution facilities. It is suggested that the integration among all the meat industry stakeholders is quite essential for the sustainability of the industry's supply chain to cope with such devastating conditions the future may hold. This review aimed to discuss different aspects of the meat industry and supply chain during the COVID-19 pandemic and proposed some future directions.

## Introduction

The COVID-19 pandemic adversely affected many sectors of life, taking a huge toll not only on the economy but the livestock industry, such as global meat production and supply chain. Several countries' preventive measures included travel restrictions, border controls, and country lockdowns, developed harsh consequences affecting production and supply chain. Meat production and processing were compromised due to difficulty of purchasing production inputs such as feed for animals, restrictions of transportation of live animals including seasonal border crossing restrictions, accessing professional services and workforce, and restrictions in supplying meat and meat products to the markets (1–3). These problems caused a drop in capacity for meat production and plants' processing, resulting in decreased sales conditions that slowed down market activity (4). Furthermore, during this COVID-19 pandemic, there was a decrease in governmental capacities to prevent, control and treat animal diseases. This was mainly due to the reallocation of the resources needed to respond to the pandemic effectively. Particularly, prevention and control of transboundary diseases such as Foot and Mouth Disease, African Swine Fever, Avian Influenza, and other infectious animal diseases have been severely compromised meat production and supply chain (5).

The COVID-19 virus rapidly spread among meat plant workers due to prolonged contact with infected co-workers, inability to follow social distancing at the workplace, shared working areas, and common transportation methods to and from work. These infected workers caused the spread of illness at community levels (6). Therefore, many plants began to close temporarily to stop the spread of illness on a larger scale. The closing of these facilities contributed to the sharp decline in the supply chain, leading to decreased meat production capacities (7). The production capacity loss reached 25–43% for beef slaughterhouses in the United States (8).

The prices of meat and meat products also fluctuated due to a gap in demand and supply, mainly due to panic buying and lockdown restrictions. Therefore, this review aimed to highlight the impact of COVID-19 on meat production, supply chain, and price fluctuations. This will be helpful to formulate future recommendations to ensure a stable meat supply chain. It will also help livestock farmers, animal health professionals, slaughterhouse workers, meat processors, traders, and policymakers to effectively combat worse situations in the future.

## Origin of COVID-19

The SARS-CoV-2 is a newly identified virus that caused the outbreak of pneumonia in Wuhan, China, in December 2019. Within no time, SARS-CoV-2 was isolated on January 7, 2020, and complete sequencing was performed. Initially, the World Health Organization (WHO) named the coronavirus as the 2019-novel coronavirus (COVID-19) on January 12, 2020. It was reported that the genome sequence of COVID-19 is quite similar to a bat CoV RaTG13 and has 96.2% identity and 79.5% similarity to SARS-CoV as well. After studying the genome sequence results and evolutionary analysis, bats were assumed to be the virus's natural hosts. Additionally, it was also assumed that SARS CoV-2 might be transmitted from bats via unknown intermediate hosts and finally infecting the human race (9).

**Figure 1:**
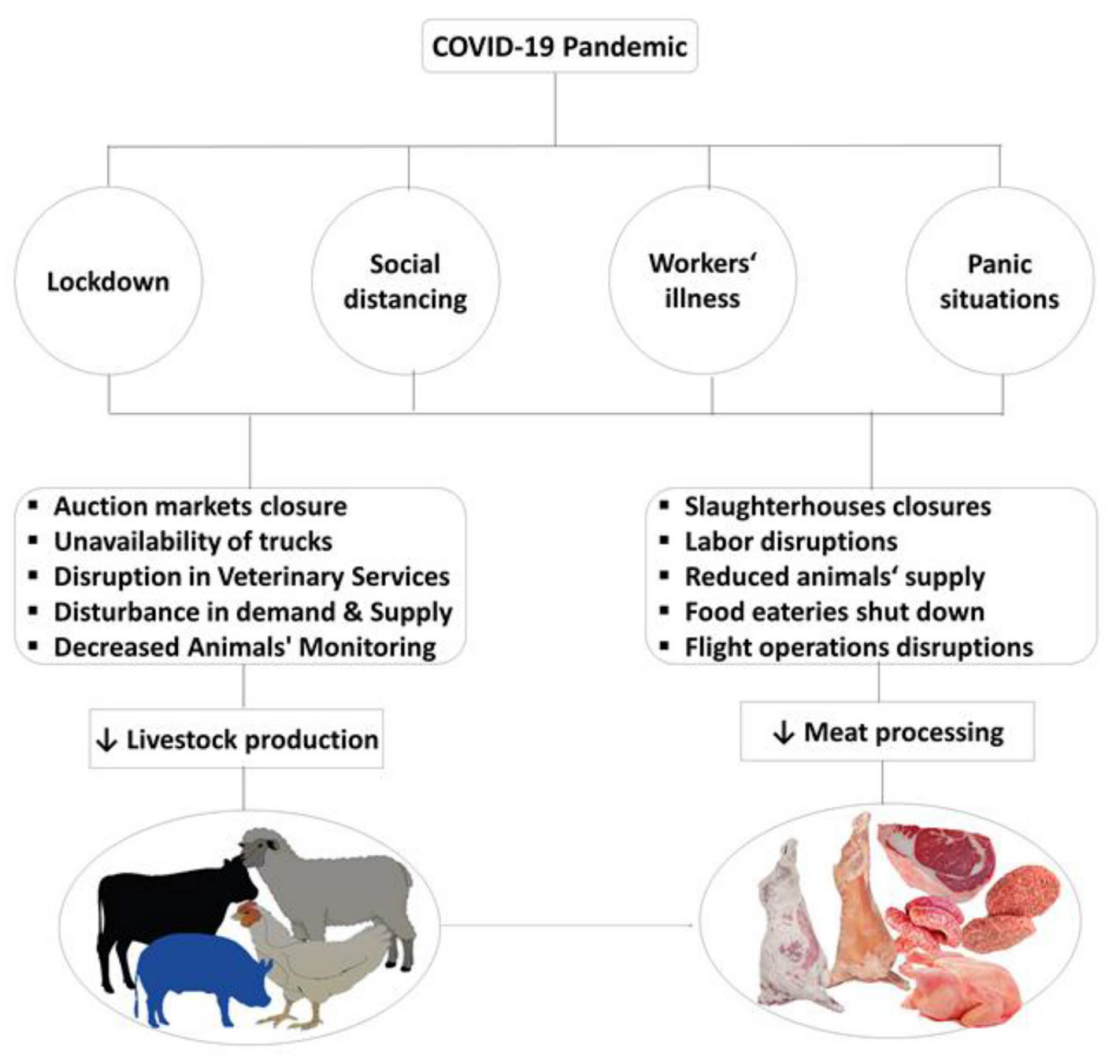
Impact of COVID-19 on meat production and supply chain.

On January 5, WHO warned the world about implementing travel restrictions from China due to this pandemic. The first death was reported on January 11 due to COVID-19. The infamous COVID-19 was declared a public health emergency on January 30, 2020, due to its rapid spread and severity. It was declared as a pandemic on March 11, 2020, by WHO. The most common signs shown by affected patients with COVID-19 were fever, cough, and rare gastrointestinal infection (10). After the outbreak of COVID-19 in Wuhan, it rapidly spread worldwide and affected all sectors of life. Meat industries also severely affected and disrupted production and supply chain. The impact of COVID-19 on meat production and supply chain is shown in Figure 1.

## Impact of COVID-19 on Meat Production

The COVID-19 pandemic has directly and indirectly impacted overall meat production. Through the infected workforce causing the shutdown of meat plants, there was a decrease in production, processing, distribution, and marketing potential (8). Farmers faced difficulty when searching for a more suitable market to sell their animals. The sale of expensive primal meat cuts was decreased due to the temporary shutdown of food eateries which affected the income coming in from meat and meat products (11). Furthermore, the decreased income status of consumers also affected meat production (12). During these circumstances, a decrease in meat production went from 338.9 million tons (carcass weight) in 2019 to 333.0 million tons in 2020 (5, 13). The pandemic's initial estimated impact on the beef industry is around $13.6 billion, with additional influences that can occur in the future (14).

The short-term impact of COVID-19 on Spanish small ruminant flock production was investigated (15). To which, the authors observed the sudden impact on meat markets, particularly in small ruminant flocks. The observations depicted a 25.9% drop in slaughtering activity of lambs and 28.5% for goats in April 2020 compared to the slaughtering data of April 2019. On the other hand, the slaughtering activity for sheep and goat kids was higher in March 2020 compared with March 2019. In another study, researchers found significant differences in the pandemic's comparative influence in different classes (steer, heifer, dairy cows, beef cows, bulls, and hogs) of animals and slaughtering volumes in different regions of the US. In May 2020, steer and heifer slaughtering volumes were 41% lower when comparing to May 2019. The biggest hit was observed in the last week of April and the first week of May 2020; the processing numbers for pork and beef declined by 40% compared to the same periods of 2019. During an 8-week study starting April 5th, cattle slaughtering was reduced by 1.14 million head, which is 22% lower than the prior year. There was a 13% reduction (2.36 million head) during those 8 weeks compared to 2019 (16) for pork. It was reported that North American meat processors that had to deal with this abnormal situation were committed to paying the extra money to retain slaughterhouse workers ($2 per hour premium and $500 bonus to all workers capable of completing consecutive shifts for 8 weeks) and attract livestock farmers to maintain the supply of animals (17). The overall global meat production and trade percentage year-on-year is given in Table 1.

**Table 1:** World meat production, trade and percentage year-on-year (Y-O-Y) change.

	**2018**	**2019**	**2020**	**Y-O-Y change**
		**Million tons**		**%**
**Production**	342.2	338.9	333.0	−1.7%
Bovine meat	71.5	72.6	72.0	−0.8%
Poultry meat	127.3	133.6	136.8	2.4%
Pig meat	120.9	109.8	101.0	−8.0%
Ovine meat	15.8	16.0	16.2	0.9%
**Trade**	33.8	36.1	37	2.4%
Bovine meat	10.5	11.2	11.1	−1.0%
Poultry meat	13.5	13.9	13.8	0.3%
Pig meat	8.4	9.5	10.6	11.2%
Ovine meat	1.0	1.0	1.0	−2.9%

*[Source: (18)]*.

### Concerns of Meat Producers

The impact of COVID-19 on livestock production was unexpected. Due to this pandemic, meat packaging facilities also closed or reduced working hours, affecting processing capacity throughout the country. Many animals could not be processed, which resulted in huge losses to the producers. Poultry processors were directed to depopulate their sheds. Most of the meat processing plants were forced to work at reduced levels than their average capacity; in the US, pig, and cattle slaughter reduced by about 40% in April 2020 compared to April 2019. There was a positive shift for mid-sized and small slaughterhouses and processing plants that ensured the availability of regional and local meat and poultry supplies during a pandemic. Unfortunately, at the same time, harvest facilities at smaller levels were insufficient to meet the demand from farmers or breeders that now have no market due to extensive plant closures, even while small plants function at maximum capacity (19).

The American Farm Bureau Federation conducted a survey that revealed an 84% of Americans hoped the government would provide financial assistance to farmers to keep them motivated during the pandemic (20). In India, farmers are no more unfamiliar with this type of emergency, but the current disturbance done by this virus outbreak is unmatched. The poultry sector that employs 1.5 million smallholder farmers observed the worst hit. Broiler meat demand fell impulsively on reports associating them to COVID-19. In the early lockdown stage, all trades regarding the meat industry in the country were distorted. This distortion impacted livestock farmers and the poultry industry, to which both need a continuous cash flow for feeding their animals (21).

In developing countries like Pakistan, farmers could not move from villages to cities due to transportation methods' unavailability. They could not sell their animals in markets due to lockdown restrictions. Initially, the meat industry and associated slaughterhouses completely shut down. Even the cattle markets were not allowed to sell the animals for slaughter at the local level. The shutting down of event venues, restaurants, and a ban on large gatherings, reduced the poultry demand by 20–25%. The ambiguity coupled with the absence of a clear strategy from the government compelled farmers to sell out their birds or meat at a reduced cost and limit their losses. Besides, farmers were also unclear about the constant supply of feed for their animals and birds at commercial farms, resulting in several of them suspending purchasing hatchlings (22).

The sudden restriction and monitoring of human activities could also affect animal health due to hindrances in veterinary services' timely provision (23). These imposed restrictions on veterinary professionals, farmers, and workers' activities cause hurdles of monitor health status and the daily requirement of animals. These scenarios resulted in the problems of overstocking at many farms that compromised animals' immune systems and increased the chances of disease outbreaks. Some farmers culled their animals to reduce the animal population, which limited animal product production (8). Feedlot cattle prices suffered the most significant hit than cull cattle and feeder cattle. For instance, feedlot cattle prices fell by 16% compared to the 9% decline in feeder cattle prices.

## Impact of COVID-19 on Meat Processing

Covid-19 showed a massive impact on the global meat processing sector. Firstly, this sector is labor-intensive, which is why it can be drastically affected by workforce disruptions. Secondly, due to food eateries being shut down, a lot of storage space is required to accommodate all the meat products due to air travel restrictions for international trade, which is not possible for most affected countries. In the US, the shutdown of processing plants began on March 27, 2020, with the closing of poultry processing facilities followed by a cascade of closing of beef, poultry, and pig processing facilities over the next couple of weeks. These closings led to reduced slaughter and processing capacity and a 45% decline in pig processing, with a similar impact on other meat production species being observed (4, 24), studied the impact of a pandemic on American and Brazilian meat sectors and demonstrated that meat processing was incredibly disruptive during April and May 2020 due to the virus outbreak at slaughtering facilities resulting in an extraordinary rise in livestock prices. Beef processors experienced a decline (21% in April and 19% in May) in production as compared to January-March 2020. In June-August 2020, the production level was near the highest level observed before processing facilities shutdown. Similarly, the pork processing industry declined (18% in April and 19% in May) compared with production in January-March 2020. The USA beef and pork processing industry was declined by 40% during April and May 2020 compared to 2019. In Canada, 75% of beef processing plants were disrupted due to the shutdown of meat facilities, particularly in the Alberta province (25). In Ghana, Covid-19 harmed the industry by inputting supply issues as the country depends mainly on importing livestock from the USA, Brazil, and the European Union. Therefore, the decline in cattle, sheep, and goat numbers was 57, 61, and 64%, respectively, during the lockdown period (26).

### Concerns of Processors

The COVID-19 outbreak has affected different societies and has caused far-reaching consequences, hence the demands of serious public health interventions and measures. Similarly, meat processors have faced many problems, including illness among plant workers and the closing of meat facilities.

#### Illness Among Plant Workers

COVID-19 transmission and chances of respiratory diseases are generally higher for the people involved in congregate work atmospheres. Meat and poultry facilities have a congregate work environment. Worker safety in such settings needs a particular focus, especially during respiratory disease outbreaks (27). CDC aggregated qualitative data from risk assessment of plants in May 2020. They reported 115 meat and poultry processing plants' data indicated that COVID-19 cases were present in 19 out of 23 states of the USA compared to 9 to 27 from April 2020. A total of 4,913 (3%) of 130,578 workers were diagnosed COVID-19 positive, ranging from 0.6 to 18.2%, while 20 deaths were recorded due to the virus in 19 states of the USA by April 27, 2020 (28). A total of 129 out of 300 workers were reported COVID-19 positive at one of Portugal's leading poultry slaughterhouses (29). Outbreaks in England and Wales have also been associated with the meat processing sector in Anglesey, Merthyr Tydfil, Wrexham, and Kirklees (30). The possible reason to control the COVID-19 at meat plants was the facility barrier (structural and operational) against its control. It was challenging to maintain a six-foot distance in the working premises, practicing practical disinfection guidelines and transport conditions (31). In the future, screening workers with COVID-19 symptoms, medical leave policies, improved disinfection, use of cloth masks, increased number of vehicles, decreased passengers to vehicle ratios, changing transport to or from the plant premises to reduce virus exposure, and mitigation efforts are some practical strategies to limit the asymptomatic and pre-symptomatic virus spread to the community (32), as well as to keep the normal health status of the workers to preserve the critical infrastructure of meat and poultry industries (33).

Therefore, it is recommended to ensure a collaborative approach of public health and precautionary biosecurity measures for workers in the field of meat and poultry processing for their self-protection, improvement of food safety, and preservation of meat production at processing facilities during the pandemic in the future (34).

#### Meat Plant as a Hotspot

Slaughterhouses and meatpacking plants were major threats for spreading the COVID-19 infection throughout the pandemic (35). It is noteworthy that the socio-demographic and labor force factors involved in COVID-19 outbreaks include young workers having more chances of asymptomatic COVID-19 disease, which are housed in short congested rooms, transported on overcrowded vehicles under inadequate or non-existent hygiene measures (36). Additionally, the traveling of employees to and from far-off rural areas in the meatpacking facilities was another factor for the increased transmission of COVID-19 (37). In May 2020, half of the COVID-19 hotspots in the United States were contracted within meat processing facilities where livestock was slaughtered and packaged in the closed areas. Furthermore, the virus proliferates higher at cold temperatures and high relative humidity, which are meat processing plants' characteristics (38). Metallic surfaces retain live viruses for longer than other environments (39, 40). Workers in proximity with others require to speak loudly over the running processing line's noise, which causes the release of more droplets and disseminates them further around the community (41). Several reports have suggested that the COVID-19 virus rise surged in meatpacking plants in several different countries, including the US, Canada, Germany, Spain, Ireland, Brazil, and Australia (42).

#### Shutdowns of the Meat Plant

The impact of COVID-19 on the livestock sector, allied fields, like the food chain, and farmers was completely unforeseen. Infected cases of workforce in meat plants grew speedily to impart ruthless effects on humans and the welfare of the environment in different countries. In the United States, meat processing plants especially poultry and pig facilities shut down when they came to know about major outbreaks. The processing capacity of pigs has been reduced up to 45%. Producers or farmers were compelled to cull their healthy animals on the farms due to the lack of animal feed supply. Disposal of the carcass was also connected with possible biosecurity risks and unfavorable effects on the environment (43). The association of spread of the virus from vectors was found predominantly among large processing plants and large meatpacking companies. However, evidences were found that plant closures decreased cases and plants that received consent from the US Department of Agriculture to increase their production line reported more cases among workers (44). Due to shut down of meat plants, the production was decreased by at least 25% (45).

## Impacts of COVID-19 on Meat Supply Chains

Covid-19 has drastically affected the meat production, processing, distribution, and consumption phases of the global meat supply chain due to lockdowns and restrictions taken by various governments. Illness of the plant workers led to the suspension of meat processing and packaging plants in various countries (7, 46). This situation decreased the production capacity up to 25, 43, and 15% of beef, pork, and chicken industries, respectively (47). Furthermore, instead of slaughtering continuously at the same rate, many animals were euthanized. Concomitantly, it is expected that the economic losses to the beef industry reached $13,617,418,450 (48), which causes severe economic losses, especially to the meat producers and processors.

In Brazil, more than 2,400 slaughterhouse workers were diagnosed as COVID-19 positive from different slaughterhouses around the country. In England, various meat processing units were suspending their operations after 246 confirmed cases of COVID-19 appeared in England and Wales (7). About 1,553 confirmed cases were reported at meat processing plants in Germany. France faced a 30% reduction in staff availability in meat processing facilities due to COVID-19. Furthermore, meat processing and packaging plants have also been affected due to physical distancing rules and less labor availability. In the United States, more than 10 million hogs were eliminated from the meat supply chain from April to September 2020. This scenario created a shortage of about two million pounds in the market that was more than 7% compared to the total production of the last year (46).

The decrease in meat production, processing and supply chain resulted in higher prices of meat and meat products. Therefore, the meat purchasing ability of poor and middle-class families was reduced that contributed to food insecurity. In China, meat production was reduced due to meat facility workers' quarantine, resulting in a decreased supply chain and increased meat prices in the local Chinese market, such as the Xinfadi market in Beijing (49). Some supermarkets have limited the number of items such as beef and pork. Furthermore, the food points also stopped serving certain products such as beef burgers.

Due to COVID-19, meat producers and processors have lost access to local and international markets for selling their products. The export restrictions have disturbed global trade as well. Many Asian countries, including the Lao People's Democratic Republic, Thailand, Myanmar, and Vietnam, were unable to export their livestock and frozen meat products to China (8). Similarly, the beef and veal export from Denmark in Europe was significantly decreased due to the COVID-19. The limited meat production also impacted the distribution of meat products and the supply chain. One of the significant factors that affected meat distribution was the restricted transportation laws due to lockdowns. In the Philippines, the delay of transportation of the raw material for meat processing created the meat shortage until the ban was loosened (8). Meat and its products are mostly transported across the borders through cargo ships. The cargo charges have been significantly increased while the product price has decreased due to COVID-19. Several meat processing plants reported higher shipment costs of meat and its products (49). Live animal transportation was also affected by the restricted movements and border checks that affected the business and compromised animal welfare (50). Overall, global trade was decreased by 13–22% during the few months of the pandemic's emergence.

The restricted movements have also threatened people's livelihood, mainly in developing countries such as East Africa (8). In these countries, many livestock producers rely on the export of live animals and frozen meat to the Middle Eastern countries during specific occasions such as Ramadan and Eid-ul-Adha's religious events. Last year, on these occasions, these people were unable to access the markets for selling their animal and frozen meat due to lockdown restrictions. Pakistan also restricted the open markets for animal sales at Eid-ul-Adha, 2020. Therefore, many people choose the online platform for the sale and purchase of animals on such occasion that was new for them, which again created problems. All these situations showed an adverse effect on meat supply chains during COVID-19.

## Changes in Consumer Behavior

**Figure 2:**
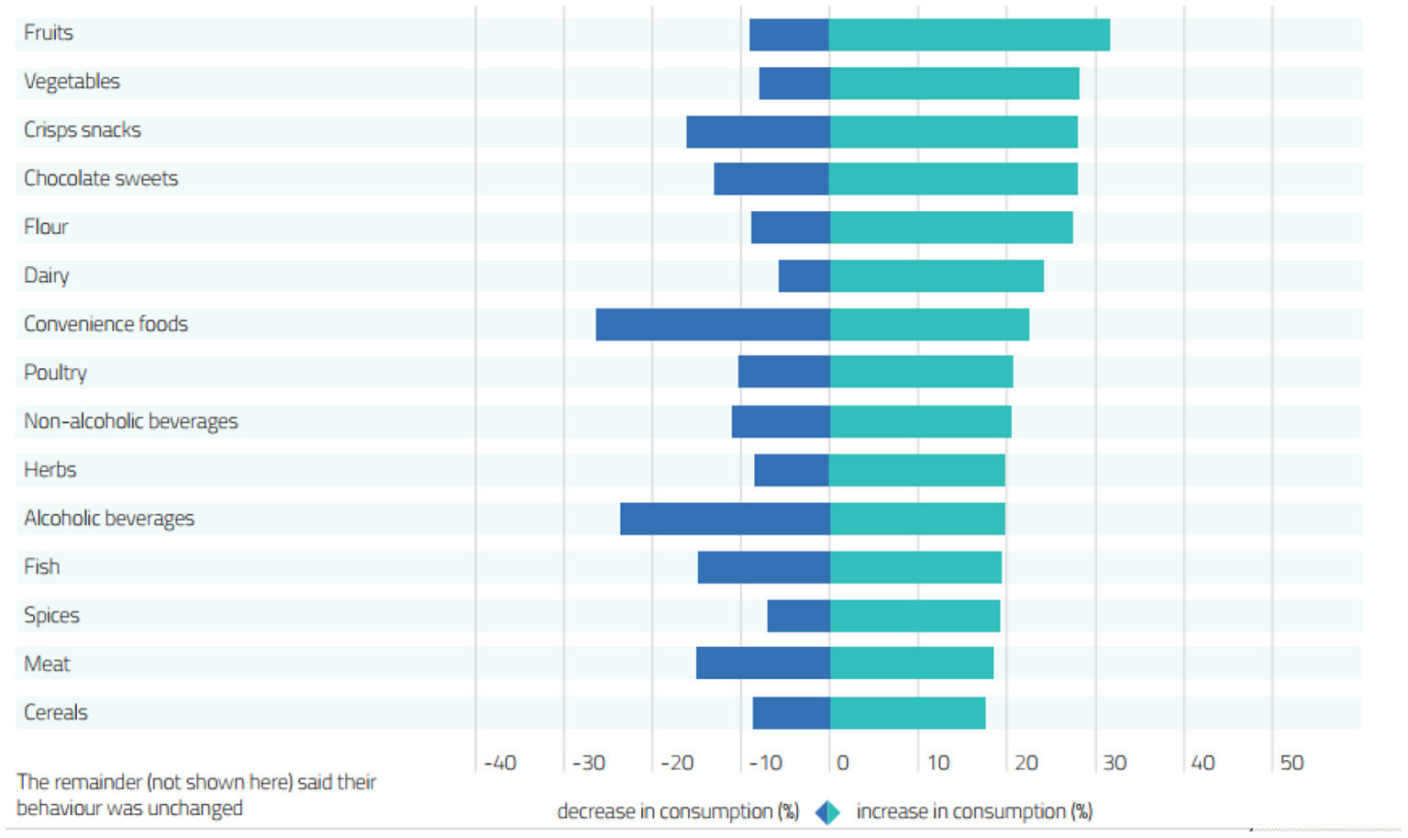
The change in product consumption by the consumers during COVID-19 pandemic [Source: (53)].

Consumers usually interact with the end product presented on the shelves of supermarkets. However, the COVID-19 pandemic has changed the purchase and consumption of meat and its products mainly due to the decreased purchasing power, staple priorities, and safety awareness among consumers. Almost half of the meat is sold in the form of food segments (educational institutes, offices, hotels, and restaurants). The closure of the educational institutes and restaurants showed an adverse effect on the supply and distribution of meat and its products (51). Furthermore, working from home has increased the consumption of meals at home that dropped the restaurants' demand. Additionally, the number of visits to superstores and spending money per visit was also changed due to COVID-19 (52). The change in different product consumption by the consumers during the COVID-19 pandemic is given in Figure 2.

The pandemic outbreak also interrupted the routine life of the people. Staying at home for longer durations resulted in higher consumptions of carbohydrates, fat, and proteins. The stress of quarantine measures pushed the people toward using more sugar-based diets for feeling motivated and positive because the food items increase the production of serotonin to combat stress (54). The demand for hamburgers increased during the pandemic as well. Higher demand for minced meat increased the price up to $4.74 per pound during the pandemic height. Similarly, pork and chicken's retail price was increased by $4.25 and 1.75 per pound, respectively.

Consumers also started to stock items that have longer shelf life during COVID-19. They also experienced the reduced availability of certain types of food items. In European countries, flour is the product that attained more attention due to home-cooking, which lead to its shortage in the market during the initial few months of the pandemic. The prices of the staple items were also increased due to the panic buying. Consumers preferred take-out or home delivery options rather than having a meal in restaurants due to lockdowns (52). Consumers found limited meat preparations in supermarkets due to pandemic. Therefore, it was time to invest in plant-based meat alternatives or in cultured meat to combat this type of situation in the future. Response rates for consumer price index for meats, poultry, and fish was decreased from 91 to 61%, and for beef and veal was declined 93–61% from June 2019 to June 2020.

## Meat Price Fluctuations

The meat products prices increased due to demand and supply issues; panic buying resulted in empty shelves. The producers were unable to supply enough products due to the closure of meat processing plants (7). Akter (55) presented that food prices in European countries significantly increased during March and April 2020, especially in countries with high restrictions, while the prices became stable in May. Pandemic had a massive impact on meat products, mainly pork's price in Beijing and Hubei provinces, which were severely hit by the virus. The peak prices were observed from January to April 2020. The highest price variations were noticed in Beijing (56). The impact of COVID-19 on price fluctuations of different meat types in different world regions is given in Table 2.

**Table 2:** Impact of COVID-19 on prices fluctuations of different meat types in different regions of the world.

**Region/country**	**Impact timing**	**Meat prices fluctuations**	**Meat type**	**References**
USA	March 6–April 10, 2020	↑39.1%	Beef Cuts (chuck, round, brisket, and loin strips)	(57)
		↓42%	Beef primal cuts (rib eye and tenderloin)	
	April 10–May 8, 2020	↑150%	Beef Cuts (chuck, round, brisket, and loin strips)	
	May2020–June, 2020	↑26.2%	Fresh beef prices	
South Carolina, USA	May, 2020	↑21.7%	All meat categories	(58)
		↑17.7%	Pork	
		↑10.5%	Chicken	
		↑100%	Ground beef	
USA	March, 2020	↑21.4% ($255/cwt in March, 2020 and $210/cwt in February, 2020)	Wholesale beef	(16)
	May 15, 2020	↑118.5% ($459/cwt on May 15, 2020)	Wholesale beef	
		↑388% ($44/cwt on May 15, 2020 and $9/cwt in February, 2020)	Wholesale pork	
Canada	April, 2020	↓40%	Pork	(59)
	September 2020	↑40%	Pork	
European Countries	March–April 2020	↑Prices	All meat categories	(55)
	May, 2020	Prices stabilized	All meat categories	
China (Beijing, Shandong, and Hubei)	January–April 2020	↑Prices	All meat categories	(56)
Indonesia	June–July 2020	↓5.93% (Rp.930/kg)	Chicken meat	(60)
Malang Regency, Indonesia	October, 2020	↓Prices	Chicken meat	(61)
Latvia	March–June 2020	↓Prices	All meat categories	(62)
Worldwide	January–December, 2020	↓7–18% (pork 17.6%, beefb10.4%, sheep 7.3%, poultry 7.0%)	All meat categories	(63)

*In table, ↑ is showing increase and ↓ is showing decrease in meat prices*.

In a recent study (57), highlighted COVID-19 impact on USA beef prices. The first wave of increase in prices was mainly due to lockdown effects starting from March 6 to April 10, 2020, up to a 39.1% increase in prices, particularly beef cuts (i.e., chuck, round, brisket, and loin strips) extensively used in ground beef production due to more demand for ground beef in the country contrarily, there was decline in primal cuts (i.e., rib eye and tenderloin) prices up to 42% due to closure of food eateries. The second wave was more pronounced due to reduced beef production from April 10 to May 8, 2020, up to a 150% increase in meat cuts prices (58), studied the meat supply chain issues in South Carolina, USA. The meat prices increased to 21.7% by the end of May 2020 due to contraction in meat supply; the increase in pork and chicken prices was 17.7 and 10.5%, respectively. The highest price jump (>100%) was observed in ground beef in May 2020 compared to previous months. In another comprehensive study about meat prices in the USA, at the end of March 2020, the wholesale prices jumped to $255/cwt compared with $210/cwt before March prices. This increase in prices was mainly due to panic buying leading to increased demand at grocery stores. The wholesale price kept increasing, reaching the highest recorded price of $459/cwt on May 15, 2020, due to a decline in meat production and shutdown of meat processing plants (16). The monthly percent changes of price indexes for meat, poultry, and other animal products from January to June 2020 is given in Table 3.

**Table 3:** The monthly percent changes of price indexes for meat, poultry, and other animal products from January to June 2020.

**Category**	**January 2020**	**February 2020**	**March 2020**	**April 2020**	**May 2020**	**June 2020**	**3-month change (March 20–June 20)**
**Export**							
Meat, poultry, and other animal products	0.1	−1.8	0.3	−4.0	5.0	−0.4	0.4
**Import**							
Meat, poultry, and other animal products	−2.3	−1.0	−4.0	−4.1	16.0	8.1	20.3
**Producer price indexes**							
Slaughter livestock	0.3	−4.4	−8.1	−3.1	10.3	−10.5	−4.4
Meats	−2.1	−2.4	−1.8	4.2	40.4	−27.7	5.7

*[Source: (64)]*.

In contrast (59), reported a decline in meat (pork) prices in Canada compared with the baseline values, starting in the last week of March 2020, the lowest prices (40% decline) were recorded in mid-April, which could be due to closure of food eateries. Subsequently, the prices started increasing; the highest prices (40% increase) were observed in Ontario during September 2020, possibly due to a decrease in meat processing. Similarly (63), found that in 2020, the international meat product prices decreased by 7–18% compared to their baseline values. It is mainly due to the slowdown of the global economy due to travel restrictions. The highest declined was observed in pork prices (17.6%), followed by beef (10.4%), sheep (7.3%), and least in poultry (7.0%). In 2021, there is an increase in pork and sheep while beef and poultry are still below their regular prices. The authors estimated that all meat product prices would be close to their baseline prices by 2025 (60), also reported chicken meat prices declined in Indonesia during June-July 2020. The lowest price, Rp.14730/kg, was recorded from March 19, 2020, to July, compared with 2019. This decrease of Rp.930/kg of meat prices was primarily due to less demand and oversupply issues (61), also studied chicken prices in Malang Regency, Indonesia. They witnessed the decline in chicken meat prices mainly due to demand and supply issues (62), discussed the COVID-19 impact on the Latvia meat sector; the export and local demand of meat products decreased from March-June 2020, leading to increased meat stocks products ultimately, a decline in prices, and ultimately, reduced revenues generations.

## Conclusions

The rapidly evolving nature of the COVID-19 virus created many problems for the meat industry. The limitations on animal exportation, logistics restrictions, and the shutting down of slaughterhouses, restaurants, and food services adversely affected all stages of the meat supply chain. Farmers were unable to find a suitable market to sell their live animals. Meat processing capacity also decreased due to the closure of the processing plants. The availability of meat and its products for the consumers was also compromised due to plant closures and panic buying, resulting in fluctuations in prices. Therefore, the following suggestions are proposed to maintain the different stages of the supply chain in order to combat such drastic situations in the future:

In the future, the following precautionary measures and practical recommendations should be followed to reduce the impact of such devastating pandemic situations and ensure continuity of meat production and supply chain: (1) livestock farmers should (i) communicate with suppliers of consumables, feed distributors and professionals such as veterinarians and meat processors to find solutions to secure inputs supply, farm services, and meat supply chain; (ii) talk with farmers associations to reach out the policymakers for getting the compulsory exemptions for transportation of feed, animals, and personnel; (ii) adopt strict precautionary and management measures at farms to avoid disease spread. (2) Professionals should (i) secure continuous services at farms to ensure animal health; (ii) up to date with the knowledge about the pandemic and aid farmers to ensure farm biosecurity. (3) Meat processors should (i) adopt online business models for a continuous supply of meat under such conditions; (ii) introduce modern automation at the plant to reduce labor involvement for sustainable meat production. (4) Policymakers should (i) develop the policies to reduce the effect of such pandemic on meat production and supply chain; (ii) communicate with the government to ensure continuous normal flow of inputs and outputs of the meat production system; (iii) review the existing animal disease prevention and control policies; (iv) take steps to control the food and meat prices in the market; (v) communicate with across the border governments to facilitate the controlled transportation or import and export of animals and farm inputs. (5) Meat scientists should look forward to developing cultured meat and plant-based alternatives to overcome food insecurity under such worst situations.

## Author Contributions

MI compiled the review materials, drafted the manuscript, contributed to the abstract, introduction, conclusions, future recommendations and involved in the review, and editing of the manuscript. MY contributed to the impacts of COVID-19 on meat supply chains, changes in consumer behavior, and contributed to review and editing of the manuscript. IB contributed to impact of COVID-19 on overall meat production and contributed to review and editing of the manuscript. SA contributed to the illness among workers, meat plant as hotspot, and contributed to review and editing of the manuscript. MSI wrote the meat price fluctuations, and contributed to review and editing of the manuscript. MJ and ZH involved in writing-review of the first draft and contributed in revision of the manuscript. AS contributed to shutdowns of meat plants, concerns of farmers and processors, and contributed to review and editing of the manuscript. SU contributed to the origin of COVID-19 and contributed to review and editing of the manuscript. DG-R helped to improve the grammar, sentence structures of the manuscript, and contributed to review and editing of the manuscript. All authors contributed to the article and approved the submitted version.

## Conflict of Interest

The authors declare that the research was conducted in the absence of any commercial or financial relationships that could be construed as a potential conflict of interest.
